# *Aspergillus sydowii* and Other Potential Fungal Pathogens in Gorgonian Octocorals of the Ecuadorian Pacific

**DOI:** 10.1371/journal.pone.0165992

**Published:** 2016-11-30

**Authors:** M. Mar Soler-Hurtado, José Vladimir Sandoval-Sierra, Annie Machordom, Javier Diéguez-Uribeondo

**Affiliations:** 1 Departamento de Biodiversidad y Biología Evolutiva, Museo Nacional de Ciencias Naturales (MNCN-CSIC), Madrid, Spain; 2 Departamento de Biodiversidad y Ecología de Invertebrados Marinos, Facultad de Biología, Universidad de Sevilla, Sevilla, Spain; 3 Departamento de Micología, Real Jardín Botánico CSIC, Madrid, Spain; Uppsala University, SWEDEN

## Abstract

Emerging fungal diseases are threatening ecosystems and have increased in recent decades. In corals, the prevalence and consequences of these infections have also increased in frequency and severity. Coral reefs are affected by an emerging fungal disease named aspergillosis, caused by *Aspergillus sydowii*. This disease and its pathogen have been reported along the Caribbean and Pacific coasts of Colombia. Despite this, an important number of coral reefs worldwide have not been investigated for the presence of this pathogen. In this work, we carried out the surveillance of the main coral reef of the Ecuadorian Pacific with a focus on the two most abundant and cosmopolitan species of this ecosystem, *Leptogorgia* sp. and *Leptogorgia obscura*. We collected 59 isolates and obtained the corresponding sequences of the Internal Transcribed Spacers (ITS) of the ribosomal DNA. These were phylogenetically analyzed using MrBayes, which indicated the presence of two isolates of the coral reef pathogen *A*. *sydowii*, as well as 16 additional species that are potentially pathogenic to corals. Although the analyzed gorgonian specimens appeared healthy, the presence of these pathogens, especially of *A*. *sydowii*, alert us to the potential risk to the health and future survival of the Pacific Ecuadorian coral ecosystem under the current scenario of increasing threats and stressors to coral reefs, such as habitat alterations by humans and global climate change.

## Introduction

Coral reefs are considered one of the most biologically diverse ecosystems in the marine realm [1]. They maintain a high biomass and abundance of varied organisms [2] and provide a plethora of micro-habitats to support enormous biodiversity [3–6]. In recent decades, coral reefs have experienced increasing pressures, and are disturbed by a combination of direct human impacts, *e*.*g*., habitat fragmentation and reduction of functional diversity [7], and global climate change, *e*.*g*., increasing ocean acidification and temperature, coral bleaching, etc. [8]. These conditions make reefs more susceptible to the proliferation and development of opportunistic organisms, which take advantage of the weakened corals [9,10].

The coral disease *aspergillosis* has produced significant deterioration and partial and massive mortalities of coral communities in the Caribbean Sea [11–15]. The responsible pathogen is the ascomycetous fungus *Aspergillus sydowii* (Bainier and Sartory, 1926). The first report of this disease in gorgonians dates back to 1995 [14,15], although similar symptoms and outbreaks had been previously reported in the 1980s [16]. The ascomycete fungus *A*. *sydowii* is globally distributed and occurs in diverse environments where it survives as a soil decomposing saprotroph [17–19]. It is apparently a terrestrial fungus, but it is salt tolerant and capable of growing in the sea [20]. Moreover, *A*. *sydowii* has been reported as a food contaminant [21], and a human pathogen in immune-compromised patients [22,23]. In marine ecosystems, *A*. *sydowii* has been isolated from some gorgonian communities of the Caribbean [11,24], Colombian Pacific coasts [25], and environmental samples of the Australian coastal waters [20].

Aspergillosis causes selective mortality of large sea fans [26], and suppression of reproduction in infected individuals [27]. As a consequence, coral population levels decrease [28]. The symptoms include purpling of the tissue, galling, and lesions [11], associated with necrotic sea fan tissue [14]. Prevalence (percentage of fans infected) and disease severity (mean percentage of fan tissue affected by disease) are positively correlated with water depth, and large sea fans are more likely to be infected than small fans [15,29]. Although the origin of this disease and its epidemiology is unknown, microsatellites and phylogenetic studies reveal a pattern of global panmixia among isolates. Moreover, sea isolates are interspersed with those isolated from environmental samples [30]. *Aspergillus sydowii* was isolated, identified and inoculated as the causative agent of the sea fan disease (Koch’s postulates) by previous authors [11,19]. The incidence of this pathogen can be similar to other fungal species, *i*.*e*., *Fusarium keratoplasticum* and *F*. *falciforme*, in other animals and ecosystems [31], exacerbated by the effects of global climate change and habitat alteration by humans.

In the Ecuadorian Pacific, there are no records of *A*. *sydowii* and coral reefs appear to be healthy. Due to the current trend of expansion of fungal infections and the endangered situation of coral reefs, we performed a survey in the Machalilla gorgonian gardens, which includes the most representative gorgonian species in a hot spot of marine biodiversity in Ecuador. We investigated the presence of *A*. *sydowii* in these organisms.

## Material and Methods

### Sampling

Gorgonian octocoral colonies were collected by SCUBA diving from rocky bottoms located in The Frailes, Machalilla National Park (Manabí, Ecuador) (1°30'14"S 80°48'33”W). The authority who issued the permission for each location was the "Ministerio del Ambiente, Manabí (ECUADOR)" (Permit Number: N° 016 –RM–DPM–MA). Due to the absence of symptoms, we randomly selected 40 colonies from the two most abundant and cosmopolitan gorgonian species of this area (pers. obs.), *Leptogorgia* Milne-Edwards and Haime, 1857 [32]: *Leptogorgia obscura* Bielschowsky, 1929 [33], and *Leptogorgia* sp. (under description). The colonies were collected within a range of 10 to 15 m in depth. Samples were kept in individual sterile plastic bags and processed in the laboratory under axenic conditions.

### Fungal isolation

From each colony, fragments of ca. 3 cm wide from randomly selected areas were excised using a sterile scalpel. To remove fungi not associated with the octocorals, the selected fragments were surface-sterilized with 70% ethanol for 30 s [25]. For fungal isolations, the selected fragments were transferred onto a peptone glucose agar media (PGA) [34] supplemented with penicillin (100 mg/l). In order to avoid any possible errors in the identification of coral fungi (negative control) the sea water sample was isolated. A glass-ring technique was used for isolation following the methodology described in [31]. Resulting pure cultures were maintained in PGA at 4°C. Cultures were labeled as ASP001 through ASP059 in the culture collection of the Real Jardín Botánico, Madrid, Spain (Table 1).

**Table 1:** Fungal isolates from gorgonians *Leptogorgia obscura* and *Leptogorgia* sp. from the Eastern Pacific of Ecuador and the resulting molecular identification based on phylogenetic analysis.

RJB number	Isolate number	Gorgoniidae Species	Fungus Species	GenBank Acc. Num.
ASP001	GORG01	*Leptogorgia* sp.	*Pyrenochaetopsis leptospora*	KX712403
ASP002	GORG02	*Leptogorgia* sp.	*Nigrospora* sp.	KX712404
ASP003	GORG03	*Leptogorgia* sp.	*Penicillium chrysogenum*	KX712405
ASP004	GORG04	*Leptogorgia* sp.	*Penicillium chrysogenum*	KX712406
ASP005	GORG05	*Leptogorgia* sp.	*Penicillium chrysogenum*	KX712407
ASP006	GORG07	*Leptogorgia obscura*	*Tritirachium* sp.	KX712408
ASP007	GORG08	*Leptogorgia* sp.	*Penicillium chrysogenum*	KX712409
ASP008	GORG09	*Leptogorgia obscura*	*Penicillium chrysogenum*	KX712410
ASP009	GORG10	*Leptogorgia* sp.	*Penicillium chrysogenum*	KX712411
ASP010	GORG11	*Leptogorgia obscura*	*Penicillium chrysogenum*	KX712412
ASP011	GORG12	*Leptogorgia obscura*	*Fusarium longipes*	KX712413
ASP012	GORG13	*Leptogorgia* sp.	*Penicillium chrysogenum*	KX712414
ASP013	GORG16	*Leptogorgia* sp.	*Nigrospora sp*.	KX712415
ASP014	GORG17	*Leptogorgia obscura*	*Lasiodiplodia pseudotheobromae*	KX712416
ASP015	GORG18	*Leptogorgia obscura*	*Phoma* sp.	KX712417
ASP016	GORG21	*Leptogorgia obscura*	*Penicillium chrysogenum*	KX712418
ASP017	GORG22	*Leptogorgia* sp.	*Penicillium chrysogenum*	KX712419
ASP018	GORG24	*Leptogorgia obscura*	*Fusarium longipes*	KX712420
ASP019	GORG25	*Leptogorgia obscura*	*Cladosporium dominicanum*	KX712421
ASP020	GORG26	*Leptogorgia obscura*	*Aspergillus sydowii*	KX712422
ASP021	GORG27	*Leptogorgia obscura*	*Aspergillus sydowii*	KX712423
ASP022	GORG29	*Leptogorgia obscura*	*Penicillium chrysogenum*	KX712424
ASP023	GORG30	*Leptogorgia* sp.	*Penicillium chrysogenum*	KX712425
ASP024	GORG31	*Leptogorgia obscura*	*Nigrospora* sp.	KX712426
ASP025	GORG32	*Leptogorgia* sp.	*Aspergillus wentii*	KX712427
ASP026	GORG33	*Leptogorgia* sp.	*Penicillium chrysogenum*	KX712428
ASP027	GORG34	*Leptogorgia* sp.	*Penicillium chrysogenum*	KX712429
ASP028	GORG35	*Leptogorgia obscura*	*Penicillium chrysogenum*	KX712430
ASP029	GORG36	*Leptogorgia* sp.	*Penicillium chrysogenum*	KX712431
ASP030	GORG37	*Leptogorgia* sp.	*Penicillium chrysogenum*	KX712432
ASP031	GORG38	*Leptogorgia* sp.	*Cladosporium sphaerospermum*	KX712433
ASP032	GORG40	*Leptogorgia obscura*	*Nigrospora* sp.	KX712434
ASP033	GORG42	*Leptogorgia obscura*	*Fusarium longipes*	KX712435
ASP034	GORG43	*Leptogorgia obscura*	*Penicillium chrysogenum*	KX712436
ASP035	GORG44	*Leptogorgia obscura*	*Penicillium chrysogenum*	KX712437
ASP036	GORG45	*Leptogorgia* sp.	*Penicillium chrysogenum*	KX712438
ASP037	GORG46	*Leptogorgia obscura*	*Penicillium chrysogenum*	KX712439
ASP038	GORG47	*Leptogorgia* sp.	*Penicillium chrysogenum*	KX712440
ASP039	GORG48	*Leptogorgia obscura*	*Nigrospora* sp.	KX712441
ASP040	GORG49	*Leptogorgia obscura*	*Fusarium longipes*	KX712442
ASP041	GORG50	*Leptogorgia* sp.	*Penicillium chrysogenum*	KX712443
ASP042	GORG51	*Leptogorgia* sp.	*Penicillium chrysogenum*	KX712444
ASP043	GORG53	*Leptogorgia* sp.	*Aspergillus ochraceopetaliformis*	KX712445
ASP044	GORG54	*Leptogorgia obscura*	*Nigrospora* sp.	KX712446
ASP045	GORG55	*Leptogorgia obscura*	*Penicillium chrysogenum*	KX712447
ASP046	GORG63	*Leptogorgia* sp.	*Cladosporium sphaerospermum*	KX712448
ASP047	GORG68	*Leptogorgia* sp.	*Penicillium chrysogenum*	KX712449
ASP048	GORG71	*Leptogorgia obscura*	*Capnobotryella* sp.	KX712450
ASP049	GORG76	*Leptogorgia* sp.	*Cladosporium sphaerospermum*	KX712451
ASP050	GORG77	*Leptogorgia* sp.	*Cladosporium sphaerospermum*	KX712452
ASP051	GORG78	*Leptogorgia* sp.	*Curvularia* sp.	KX712453
ASP052	GORG80	*Leptogorgia* sp.	*Alternaria* sp.	KX712454
ASP053	GORG83	*Leptogorgia obscura*	*Aspergillus sclerotiorum*	KX712455
ASP054	GORG84	*Leptogorgia obscura*	*Aspergillus sclerotiorum*	KX712456
ASP055	GORG85	*Leptogorgia* sp.	*Nigrospora* sp.	KX712457
ASP056	GORG87	*Leptogorgia* sp.	*Cladosporium sphaerospermum*	KX712458
ASP057	GORG88	*Leptogorgia* sp.	*Penicillium mallochii*	KX712459
ASP058	GORG90	*Leptogorgia obscura*	*Cladosporium dominicanum*	KX712460
ASP059	GORG92	*Leptogorgia* sp.	*Tritirachium* sp.	KX712461

### DNA extraction, PCR amplification, sequencing, and species identification

DNA was extracted from 20 mg of the fungal isolate tissues using the DNeasy extraction kit (Qiagen, Inc.) according to the manufacturer’s protocol. DNA fragments containing internal transcribed spacers ITS1 and ITS2, including 5.8S, were amplified and sequenced with primer pair ITS5/ITS4 [35]. The PCR profile was: 2.5 μl 10 x buffer, 1.4 μl 50 mM MgCl_2_, 1.6 μl 25 mM dNTPs, 0.5 μl of each 10 mM primer (forward and reverse), 1 μl 1 mg/ml BSA, 1 μl DNA, 0.3 μl 5 U/μl Taq polymerase, and 16.2 μl ddH_2_O. The PCR conditions were 1 min at 95°C, 35 cycles of 1 min at 95°C, 45 s at 58°C and 1 min at 72°C, and finally 10 min at 72°C. The amplicons were sequenced for both strands using BigDye Terminator in an ABI 3730 genetic analyzer (Applied Biosystems).

The sequences were edited and primers trimmed using the Sequencher v.4.9 program (Gene Code Corporation, Ann Arbor, MI, USA). BLAST [36] was used to compare the sequences against those existing in the National Center of Biotechnology Information (NCBI) nucleotide databases.

**Fig 1:**
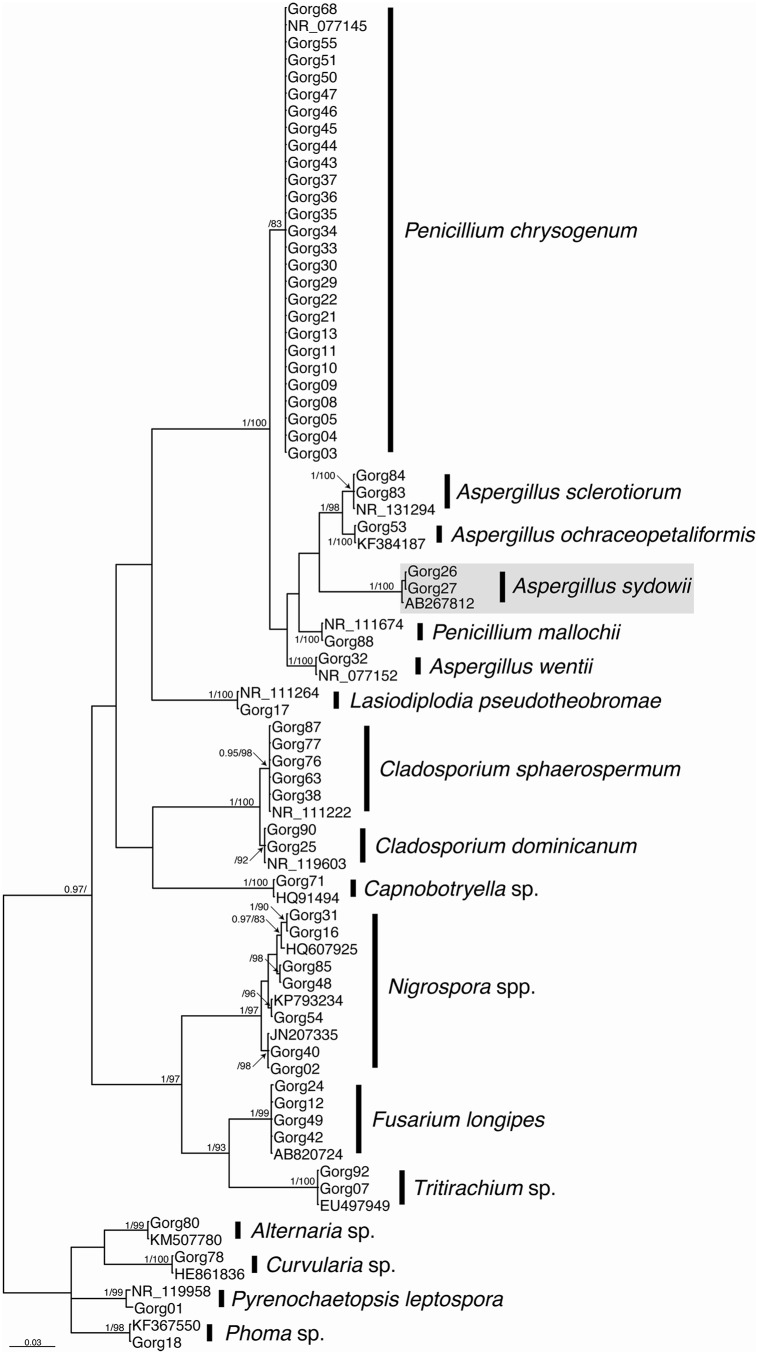
Bayesian out-group-rooted cladogram inferred from ITS rRNA gene sequences of fungal isolates from *Leptogorgia obscura* and *Leptogorgia* sp. from the Eastern Ecuadorian Pacific. Numbers placed above and below the internodes are, respectively, PP and BS of the Bayesian and Maximum Likelihood analyses.

For species identification of the isolates, the corresponding ITS sequences were phylogenetically analyzed with a number of selected ITS sequences of reference of closely related fungal species obtained from the NCBI (see Table 2, Fig 1). To perform the phylogenetic analyses, a GTR + G + I substitution model was first obtained using the jModelTest v2.1.5 [37] program. This model was selected based on the Akaike Information Criterion (AIC). Bayesian inference and Maximum Likelihood analyses were performed using MrBayes v3.2.5 [38] and RaxML v8.0.0 [39], respectively. The Bayesian inference analysis was implemented with three runs of 20 million generations sampling one tree per 1000 replicates. For each run, eight Markov chain Monte Carlo (MCMC) simulations were conducted. These simulations were run until a critical value was reached for the topological convergence diagnostic lower than 0.005. Branch supports were evaluated by posterior probabilities after a burn-in of 25%. The Maximum Likelihood analysis was implemented with a random starting tree and clade support was assessed with 1000 bootstrap replicates. The Maximum Likelihood analysis was implemented in the graphical user interface raxmlGUI v1.5 [40]. The clade support was assessed with 1000 bootstrap replicates after selecting the best tree from 100 trees generated.

**Table 2:** Genbank rDNA ITS reference sequences for fungal species used in phylogenetic analysis to identify fungal isolates from Ecuadorian gorgonians.

Species	GenBank number	Isolate / strain	Type material
***Penicillium chrysogenum***	NR_077145	CBS 306.48	yes
***Aspergillus sclerotiorum***	NR_131294	NRRL 415	yes
***Aspergillus ochraceopetaliformis***	KF384187	FJ120	-
***Aspergillus sydowii***	AB267812	CBS 593.65	-
***Penicillium mallochii***	NR_111674	DAOM 239917	yes
***Aspergillus wentii***	NR_077152	ATCC 1023	yes
***Lasiodiplodia pseudotheobromae***	NR_111264	CBS 116459	yes
***Cladosporium sphaerospermum***	NR_111222	CBS 193.54	yes
***Cladosporium dominicanum***	NR_119603	-	yes
***Nigrospora oryzae***	HQ607925	ATT291	-
***Nigrospora* sp.**	KP793234	M116	-
***Nigrospora* sp.**	JN207335	P39E2	-
***Fusarium longipes***	AB820724	IFM 50036	-
***Tritirachium* sp.**	EU497949	F13	-
***Alternaria* sp.**	KM507780	311a	-
***Curvularia* sp.**	HE861836	UTHSC:08–2905	-
***Pyrenochaetopsis leptospora***	NR_119958	-	yes
***Phoma* sp.**	KF367550	4 BRO-2013	-

### Morphological characterization

Isolates corresponding to *A*. *sydowii* according to phylogenetic analysis were morphologically characterized using scanning electron microscopy, SEM (Hitachi s3000N, Real Jardín Botánico, CSIC, Madrid, Spain). Mycelia with characteristic features were fixed in 2% glutaraldehyde for 1 h, washed in distilled sterile water, and then dehydrated for 1 h in a series of ethanol (30, 50, 70, 80, 90, 95 and 100%) solutions. The isolates dehydrated in absolute ethanol were critical-point dried and the material was sputter coated in a vacuum with an electrically conductive layer of gold to a thickness of about 80 nm. Samples were observed at a beam specimen angle of 45° with an accelerating voltage of 20kV and final aperture at 200 μm.

## Results

### Fungal isolation and species identification

A total of 59 fungal isolates were obtained and the phylogenetic analyses resulted in 17 phylogenetically supported clusters (Fig 1). The majority of the isolates could be assigned to a species reference sequence (from type material). These species were: *Penicillium chrysogenum*, *P*. *mallochii*, *Aspergillus sclerotiorum*, *A*. *ochraceopetaliformis*, *A*. *sydowii*, *A*. *wentii*, *Lasiodiplodia pseudotheobromae*, *Cladosporium shpaerospermum*, *C*. *dominicanum*, *Fusarium longipes*, and *Pyrenochaetopsis leptospora*. Five groups of isolates grouped with sequences of references of unknown species: *Alternaria* sp., *Capnobotryella* sp. *Curvularia* sp., *Phoma* sp., and *Tritirachium* sp. The sequences grouping into the cluster containing *Nigrospora* spp. were diverse and could not be assigned to any known ITS sequences (Table 2).

**Fig 2:**
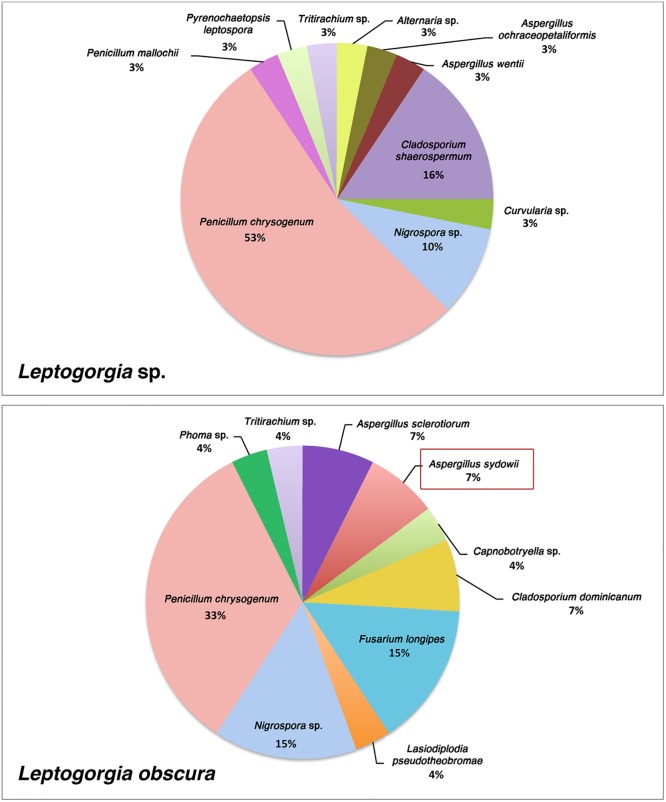
Pie charts showing isolation frequency of different fungal isolates from each host gorgonian species, *Leptogorgia obscura* and *Leptogorgia* sp. of the Eastern Ecuadorian Pacific.

The majority of the isolates belonged to the genera *Penicillium* and *Aspergillus* (Fig 2). The most frequent species was *Penicillium chrysogenum* (24 out of 59), which occurred in colonies of both *L*. *obscura* and *Leptogorgia* sp. (Fig 2), followed by *Nigorspora* spp. and *Penicillium granulatum* (both 4 out of 59 each), to *Aspergillus sydowii* (2 out of 59). The species *A*. *sydowii* and *Fusarium longipes* were only found in *L*. *obscura* (Fig 2).

### Morphological characterization

**Fig 3:**
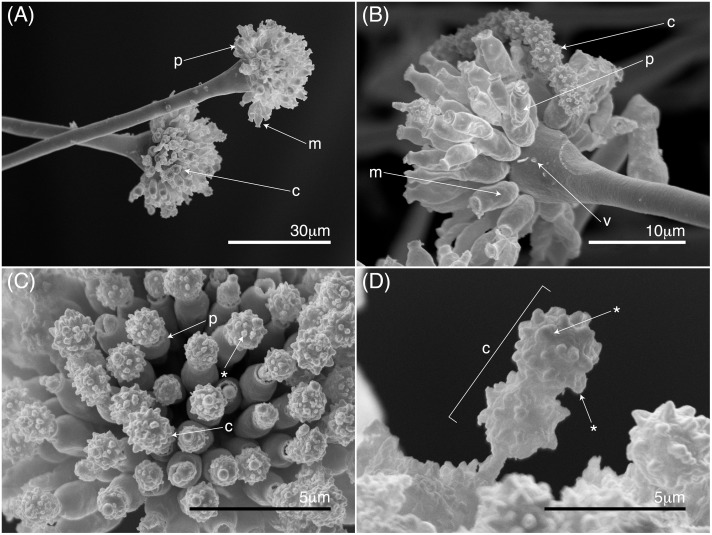
Scanning electron microscopy showing characteristic features of conidiophores of *Aspergillus sydowii* isolated from *Leptogorgia obscura* and *Leptogorgia* sp. of the Eastern Ecuadorian Pacific: (A) conidiophore structure with metule (m), phialide (p), and globose conidia (c); (B) morphological features of conidia head with a vesicle characteristic (v), metulae (m), phialide (p), and globose conidia (c); (C) phialide (p) and mature globose conidia (c) with verruculose ornamentation (*); (D) globose conidia (c) with verruculose ornamentation (*).

The isolates molecularly assigned to *A*. *sydowii* showed conidiophores with conidia, metulae, and phialides seen in the SEM micrographs (Fig 3). The length of the metulae ranged from 2.5 to 3.5 μm, and the breadth from 4.2 to 6.5 μm. Phialides ranged from 2.2 to 3.0 μm in length and from 3.4 to 6.1μm in breadth. The conidia were globose with a roughened or spinose ornamentation. These conidia had a diameter that ranged from 2.5 to 4.4 μm (Fig 3).

## Discussion

The Ecuadorian Pacific coral reefs are one of the most important and unstudied marine ecosystems and biodiversity ‘hot spots’. In this study, we found that the gorgonian communities of these reefs, specifically *L*. *obscura* and *Leptogorgia* sp. colonies, hold a large, diverse, and mostly unknown fungal community in these hosts. Interestingly, this fungal community includes the coral pathogen *A*. *sydowii*. This constitutes the first report of this pathogen in Ecuador and the second in the Eastern Pacific. The gorgonian community appeared to be healthy and showed no symptoms of fungal disease during the sampling period. These results are similar to those of [13,41], who also found isolates of *A*. *sydowii* in healthy colonies of *Gorgonia ventalina* in the Caribbean. In Ecuador, this pathogen was only found in the samples of *L*. *obscura* but not in those of *Leptogorgia* sp. This could be due to the limited sampling and the low prevalence of this fungus in these populations (only 2 specimens out of 59). A different susceptibility of the gorgonian species could also explain this result. A previous study [14] indicated differences in the incidence of aspergillosis between two species of gorgonians, *e*.*g*., *G*. *ventalina* and *G*. *flabellum*.

Moreover, we provide new data on fungal communities of marine environments, particularly in coral reefs. Studies on fungal communities in coral reefs are scarce. In Singapore, an investigation on 10 species of gorgonian corals indicated the presence of 16 fungal genera, including *Acremonium*, *Aspergillus*, *Chaetophoma*, *Cladosporium* and *Penicillium* [42]; among these genera, the species *Cladosporium sphaerospermum* and *Phoma* sp. were also found in our study. In a study carried out in colonies of the Caribbean sea fan *G*. *ventalina* [43], 15 new fungal species were found, corresponding to 8 genera, including *Aspergillus*, *Cladosporium*, *Gloeotinia* and *Penicillium*. In 2008, as part of a larger sampling effort, the same authors identified 35 fungal species corresponding to 15 genera, including *Aspergillus*, *Cladosporium*, *Nectria*, *Penicillium* and *Stachybotrys* [13]. Among these genera, the species *A*. *sydowii* and *C*. *sphaerospermum* were also found in our study.

In the Pacific, the only sampled coral reef area studied for fungal community composition was at Chocó, Colombia [25]. This area is located near the coral reef investigated in our study. They found 59 fungal species in the sea fan *Pacifigorgia* spp. corresponding to 13 fungal genera (*e*.*g*., *Aspergillus*, *Penicillium*). In our work, we only found *A*. *sydowii* and *A*. *sclerotiorum*, two species already reported in the eastern Pacific [25]. Thus, all other fungal species identified in our study represent the first report of this species in the Pacific gorgonians. We also found species that had been previously reported in gorgonians. These included *Penicillium chrysogenum* [13,25,44] and *Cladosporium sphaerospermum* [43]. However, the role of pathogens of these species remains unknown.

We found some fungal species that had never been described in gorgonians, including *Aspergillus fumigatus* (previously described in soil, air, water, food, plants and organic matter), *Capnobotryella* sp. (previously described in lichens and pumpkins), *Fusarium longipes* (previously described in soil of tropical regions), *Lasiodiplodia theobromae* (previously described causing damage in vascular plants), *Phoma* sp. (described from soil, as saprophytes on various plants, and as pathogens in plants and humans) and *Pyrenochaetopsis leptospora* (soil borne and mainly associated with gramineous plants). The pathogenicity of these fungal species to gorgonians is unknown. However, whether these species are also characteristic of marine environments or originate from land as agricultural runoff and move to marine environments via discharges from rivers is unknown.

Other species found in this work have been previously found in marine environments but not in gorgonians or coral reefs. For example, *Aspergillus ochraceopetaliformis* and *Alternaria* sp. were found in deep-sea environments [45,46], *Nigrospora* sp. in sea anemones [47], or saline environments in general, such as *Penicillium mallochii* was before isolated from the guts of tropical leaf-eating caterpillars in Costa Rica [48].

In spite of the presence of known pathogens, such as *A*. *sydowii*, and a wide diversity of fungal pathogens in the Ecuadorian coral reefs, we did not observe any damage or mortality. The factors that lead to aspergillosis or the development of other fungal pathogens on coral reefs are unknown. In phylogenetically related fungal pathogens, some environmental stressors influence the development of disease [31,49]. It was suggested that coral aspergillosis could be enhanced by abiotic factors [50,51] and speculated that this disease could be the result of specific combinations of environmental factors (*e*.*g*., humidity, UV, temperature, aerosol concentrations) or of large-scale climate patterns (oceanic currents) [50]. Specifically, the microclimatic parameter of temperature has been shown to be involved in the gorgonia-*A*. *sydowii* interaction by promoting the growth and activity of the pathogen and reducing the efficacy of host defenses [24,25].

Thus far, studies on coral diseases, such as *Aspergillosis*, are limited by the lack of isolates from marine sources prior to epidemics [30]. The detection of these pathogens in healthy Ecuadorian gorgonian gardens is, therefore, of key importance since it indicates the presence of *A*. *sydowii* and other potential coral pathogens in asymptomatic colonies. This can help in further investigations aiming to decipher the factors leading to gorgonian disease and an eventual deterioration of this ecosystem. Alert models on diverse environmental stressors and the monitoring of the health of coral reefs are crucial for a better understanding of the development of aspergillosis disease. Future studies on this pathogen and the factors triggering disease require the comparison of healthy coral reefs with the presence of *A*. *sydowii* to those affected by this pathogen.

Understanding where and to what extent potentially harmful organisms exist is of crucial importance to the design of adequate conservation plans. Fungal diseases currently represent one of the main threats to biodiversity worldwide [52] and corals are no exception. The results presented here contribute to a better understanding of the biodiversity of fungal communities in coral reefs and the construction of data-baselines for the presence and incidence of these fungal pathogens in natural systems and in particular coral reefs.
